# Supplementary material for: Inter-subject Correlation While Listening to Minimalist Music: A Study of Electrophysiological and Behavioral Responses to Steve Reich's Piano Phase
Source: Front Neurosci. 2021 Dec 9;15:702067. doi: 10.3389/fnins.2021.702067 (PMC8695499; doi:10.3389/fnins.2021.702067)
Supplement: Supplementary file 1 [file Data_Sheet_1.PDF]

Supplementary Material

1 SUPPLEMENTARY DATA

The data generated and analyzed in this study can be found in the Naturalistic Music EEG Dataset—Minimalism (NMED-M) in the Stanford Digital Repository (https://purl.stanford.edu/kt396gb0630).

2 SUPPLEMENTARY FIGURES AND TABLES

2.1 Figures

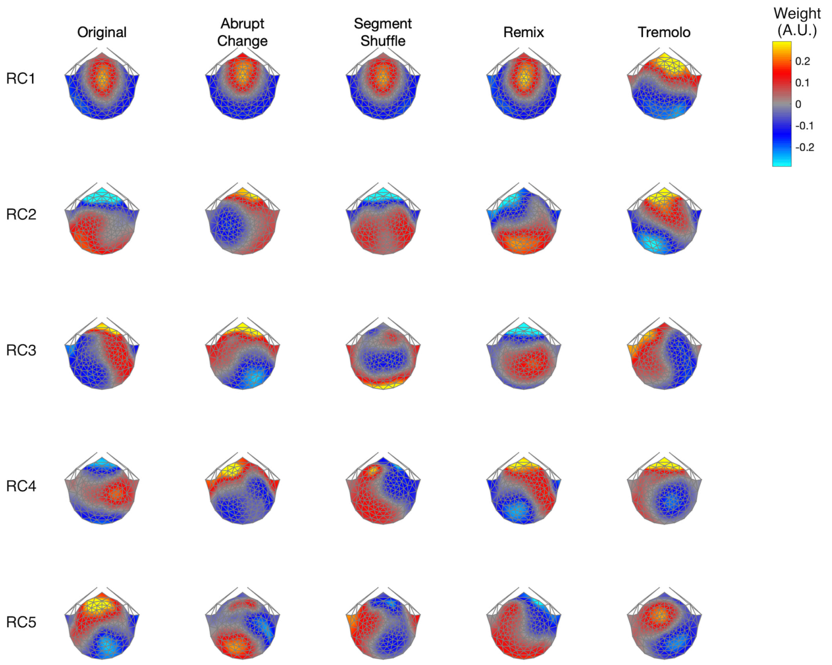

Figure S1. Reliable components (RC) 1-5 for each stimulus.

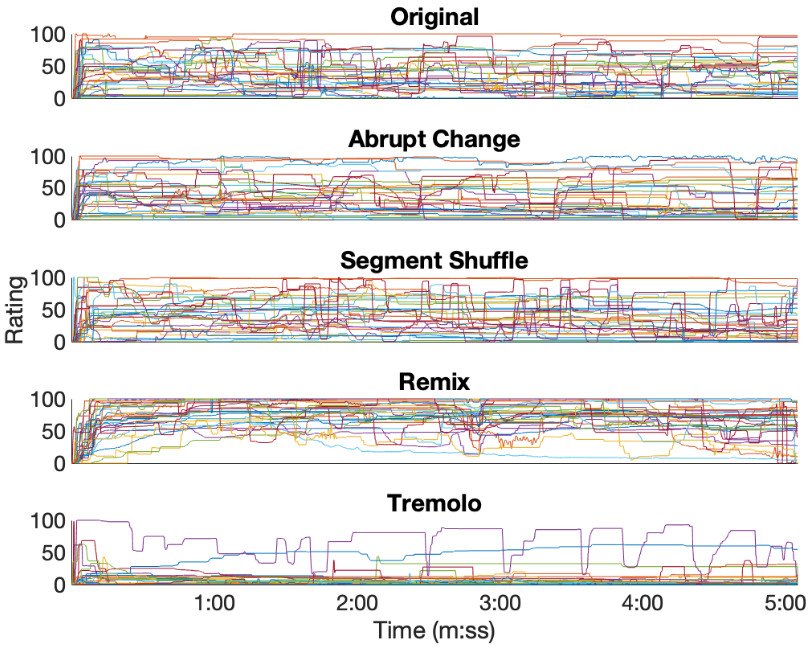

Figure S2. Continuous behavioral (CB) reports of engagement from individual participants, grouped by stimulus condition.

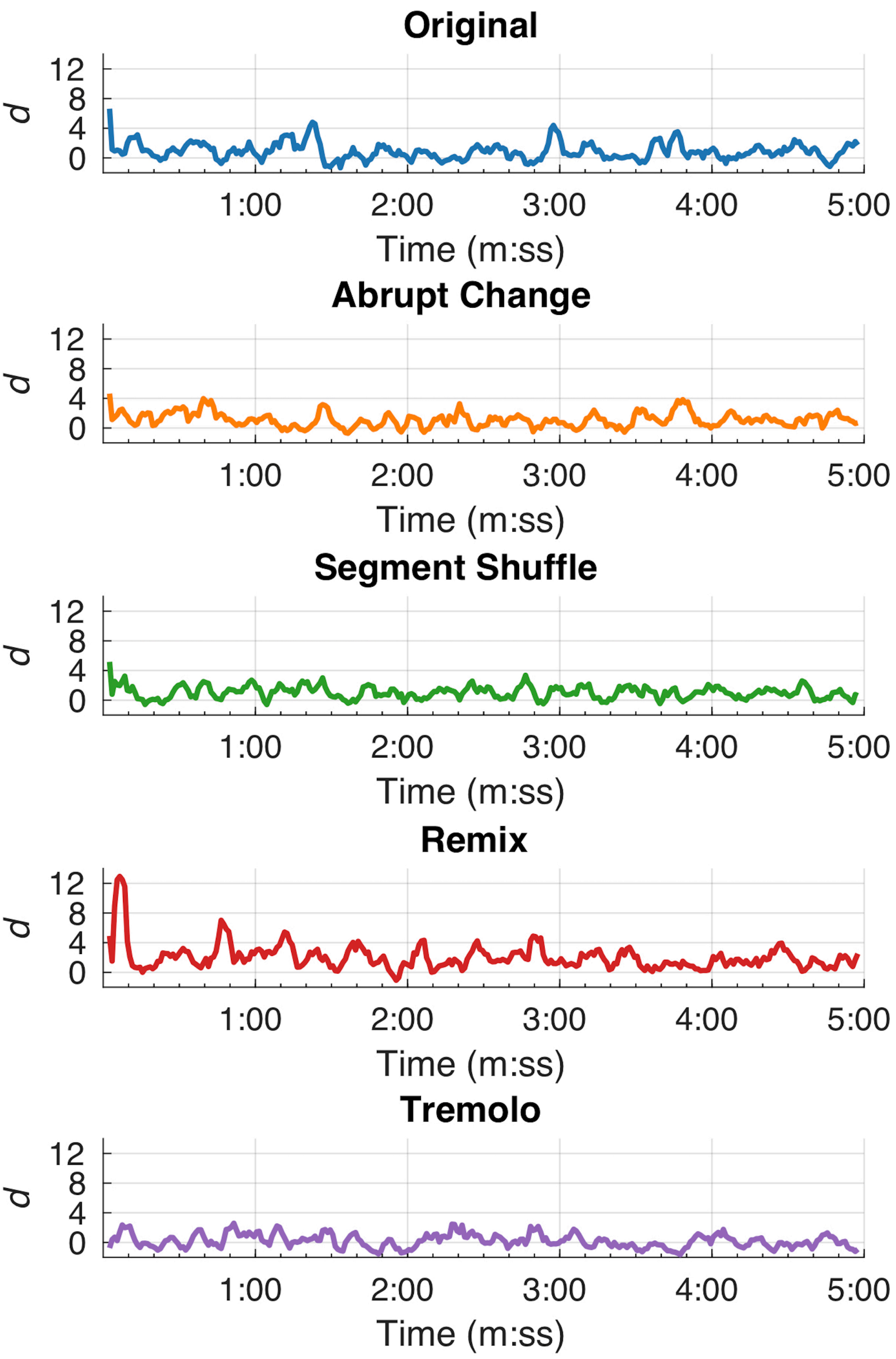

Figure S3. Time-resolved effect sizes for EEG ISC.

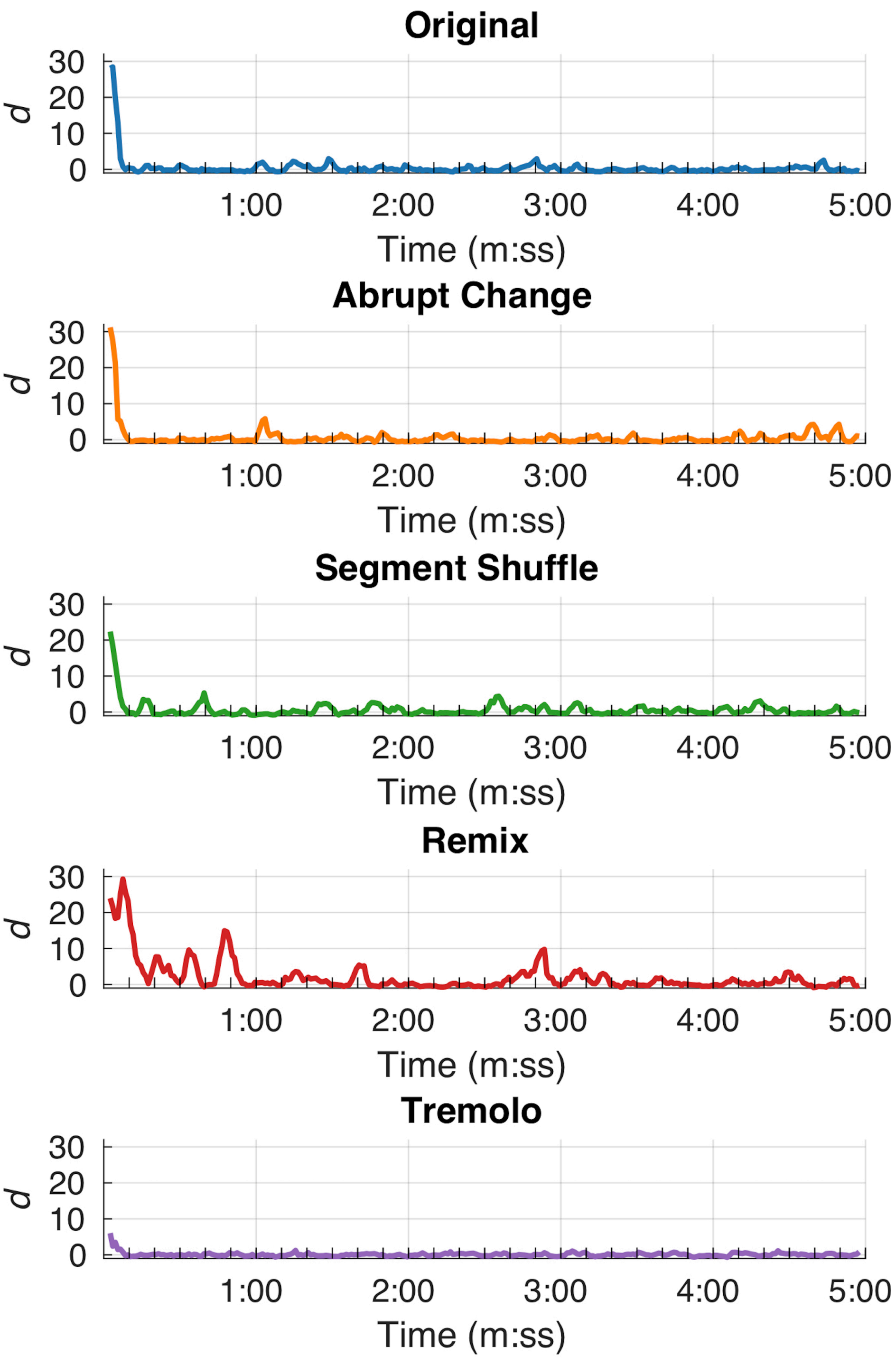

Figure S4. Time-resolved effect sizes for CB ISC.

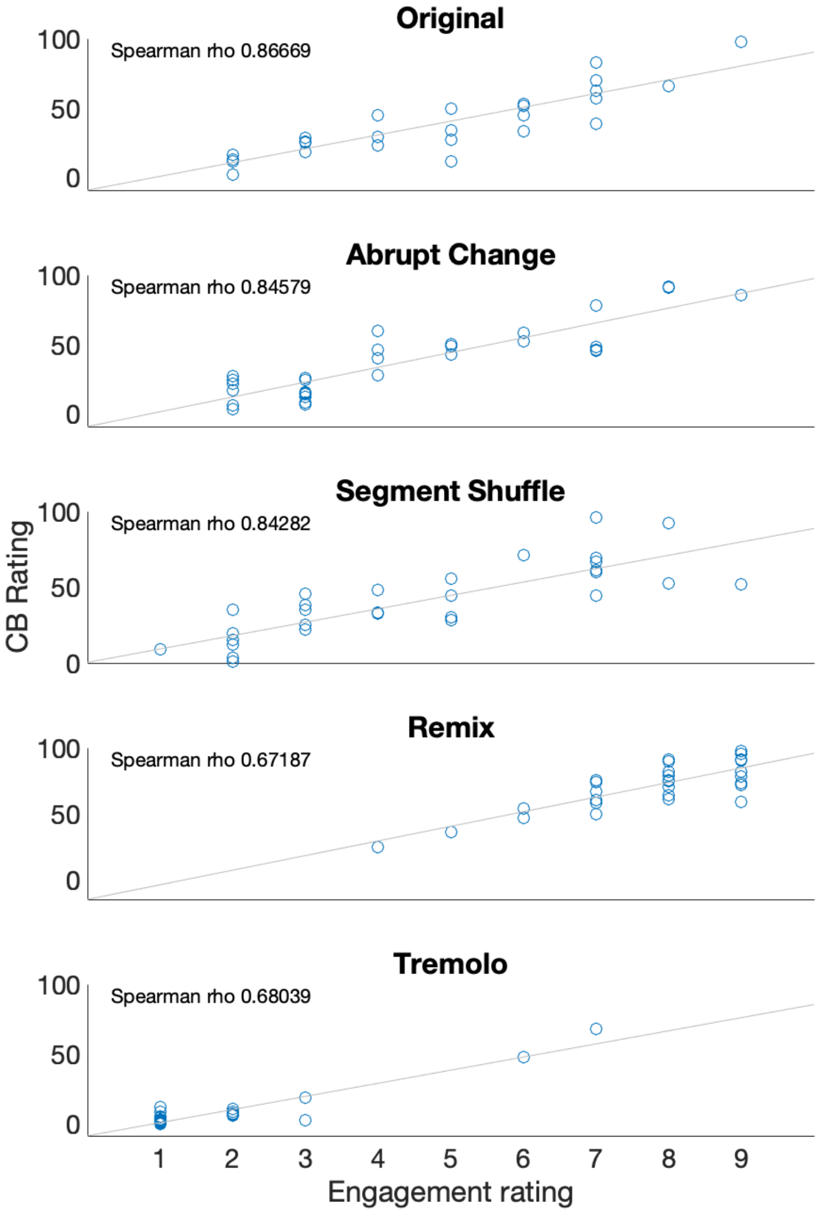

Figure S5. Scatter plot of each participant's mean CB value with the behavioral rating in response to the question "How engaging was the stimulus?" Spearman's rho also reported for each correlation.

2.2 Tables

Event number	Time (m:ss)	Event	Description
1	0:06	percussion entry	After seconds of hearing only a *Piano Phase* sample, a lead-in drum machine attack starts at 0:05 and builds into simultaneous marimba and synth countermelody entrances.
2	0:19	percussion entry	Another rhythm enters via cymbal brush.
3	0:44	percussion entry	Lead-in drum machine attack begins and culminates in the beat drop at 0:47.
4	1:10	percussion dropout & synth strings entry	Just before the percussion drops out there is a brief rhythm lick.
5	1:25	piano entry & synth strings entry	*Piano Phase* sample.
6	1:38	percussion entry	Percussion kick (i.e., lead-in attack) followed by ongoing low-pitched beat pattern.
7	2:01	percussion dropout	Temporarily only meandering synth and *Piano Phase* sample.
8	2:04	percussion entry	Return to texture from before 2:01.
9	2:18	meandering synth dropout & block percussion entry	This is the first time the block percussion enters.
10	2:30	piano entry & pitched percussion entry	A *Piano Phase* sample enters as part of a shift towards a denser texture that immediately begins to fade out.
11	2:36	all but piano drops out	A dramatic texture reduction that leaves only a *Piano Phase* sample.
12	2:50	pitched percussion entry	Part of a move towards a lusher texture.
13	3:03	meandering synth entry & cymbal brush entry	Cymbal brush as in the opening.
14	3:15	percussion entry	The start of a rhythm kick.
15	3:29	percussion dropout & synth strings entry	Dramatic texture change with percussion fade out similar to 1:10.
16	3:41	percussion entry	Rhythmic kick as at the opening and then an ongoing beat.
17	3:55	harpsichord entry	A descending pattern that recurs every few seconds.
18	4:09	piano entry	A *Piano Phase* sample.
19	4:35	synth entry	Return of the same synth line that dropped out in 4:22.

Table S1. Musical events in Remix predicted to be salient for listeners.

	**Original**	**Abrupt Change**	**Segment Shuffle**	**Remix**
**Abrupt Change**	0.848 (0.067)			
**Segment Shuffle**	0.179 (0.334)	0.139 (0.379)		
**Remix**	< 0.001∗ (1.095)	< 0.001∗ (1.213)	< 0.001∗ (1.643)	
**Tremolo**	< 0.001∗ (1.286)	< 0.001∗ (1.358)	< 0.001∗ (1.109)	< 0.001∗ (2.781)

Table S2. Post hoc t-test p-values (FDR-corrected) for ratings of how "pleasant" the stimulus was. Asterisks denote FDR-corrected p-values less than 0.05. Effect size (Cohen's D) in parentheses following each p-value.

	**Original**	**Abrupt Change**	**Segment Shuffle**	**Remix**
**Abrupt Change**	0.449 (0.344)			
**Segment Shuffle**	0.164 (0.320)	0.449 (0.162)		
**Remix**	0.003* (0.741)	< 0.001∗ (0.955)	< 0.001∗ (1.374)	
**Tremolo**	< 0.001∗ (1.841)	< 0.001∗ (1.655)	< 0.001∗ (1.546)	< 0.001∗ (3.918)

Table S3. Post hoc t-test p-values (FDR-corrected) for ratings of how "musical" the stimulus was. Asterisks denote FDR-corrected p-values less than 0.05. Effect size (Cohen's D) in parentheses following each p-value.

	**Original**	**Abrupt Change**	**Segment Shuffle**	**Remix**
**Abrupt Change**	0.719 (0.129)			
**Segment Shuffle**	0.065 (0.448)	0.036* (0.543)		
**Remix**	0.027* (0.751)	0.040* (0.611)	< 0.001∗ (1.191)	
**Tremolo**	0.036* (0.408)	0.024* (0.560)	0.719 (0.065)	< 0.001∗ (0.846)

Table S4. Post hoc t-test p-values (FDR-corrected) for ratings of how "well ordered" the stimulus was. Asterisks denote FDR-corrected p-values less than 0.05. Effect size (Cohen's D) in parentheses following each p-value.

	**Original**	**Abrupt Change**	**Segment Shuffle**	**Remix**
**Abrupt Change**	0.954 (0.018)			
**Segment Shuffle**	0.226 (0.329)	0.226 (0.340)		
**Remix**	0.002* (0.991)	0.002* (0.833)	< 0.001∗ (1.249)	
**Tremolo**	< 0.001∗ (1.242)	< 0.001∗ (1.131)	< 0.001∗ (0.980)	< 0.001∗ (2.481)

Table S5. Post hoc t-test p-values (FDR-corrected) for ratings of how "interesting" the stimulus was. Asterisks denote FDR-corrected p-values less than 0.05. Effect size (Cohen's D) in parentheses following each p-value.

	**Original**	**Abrupt Change**	**Segment Shuffle**	**Remix**
**Abrupt Change**	0.508 (0.223)			
**Segment Shuffle**	0.593 (0.155)	0.835 (0.051)		
**Remix**	< 0.001∗ (1.376)	< 0.001∗ (1.722)	< 0.001∗ (1.444)	
**Tremolo**	< 0.001∗ (1.269)	< 0.001∗ (1.051)	< 0.001∗ (0.912)	< 0.001∗ (3.135)

Table S6. Post hoc t-test p-values (FDR-corrected) for ratings of how "engaging" the stimulus was. Asterisks denote FDR-corrected p-values less than 0.05. Effect size (Cohen's D) in parentheses following each p-value.

	Original	Abrupt Change	Segment Shuffle	Remix	Tremolo
**RC1**	< 0.001*	< 0.001*	< 0.001*	< 0.001*	0.379
**RC2**	0.442	0.379	0.723	0.079	0.442
**RC3**	0.231	0.259	0.379	0.050	0.442
**RC4**	0.433	0.379	0.379	0.005*	0.379
**RC5**	0.238	0.194	0.761	0.442	0.836

Table S7. P-values (FDR-corrected) for RC coefficients. Asterisks denote FDR-corrected p-values less than 0.05.

**Abrupt Change**	0.119 (0.334)			
**Segment Shuffle**	0.210 (0.321)	0.719 (0.062)		
**Remix**	< 0.001* (1.406)	< 0.001* (0.987)	< 0.001* (1.146)	
**Tremolo**	< 0.001* (0.644)	< 0.001* (0.715)	< 0.001* (1.067)	< 0.001* (1.766)
	**Original**	**Abrupt Change**	**Segment Shuffle**	**Remix**

Table S8. Post hoc t-test p-values (FDR-corrected) for overall EEG ISC values. Asterisks denote FDR-corrected p-values less than 0.05. Effect size (Cohen's D) in parentheses following each p-value.

**Abrupt Change**	0.874 (0.056)			
**Segment Shuffle**	< 0.001* (0.512)	< 0.001* (0.571)		
**Remix**	0.010* (2.249)	0.014* (3.058)	< 0.001* (2.436)	
**Tremolo**	0.007* (0.763)	0.005* (0.699)	< 0.001* (1.067)	< 0.001* (2.881)
	**Original**	**Abrupt Change**	**Segment Shuffle**	**Remix**

Table S9. Post hoc t-test p-values (FDR-corrected) for overall CB ISC values. Asterisks denote FDR-corrected p-values less than 0.05. Effect size (Cohen's D) in parentheses following each p-value.
